# A Report of the Practice of Midwifery at the Westminster General Dispensary, during 1819

**Published:** 1822-04

**Authors:** 


### Art. VIII.—*A Report of the Practice of Midwifery at the Westminster General Dispensary, during* 1819. By the Editor.

WHEN I ventured, for the first time, to lay before the public an account of the practice of midwifery, during the first year of my performing the duties of physician-accoucheur at the Westminster General Dispensary, I expressed a hope that a publication of that kind would serve the purpose of stimulating the medical officers of other charitable institutions for the delivery of poor women, to report the result of their practice, from which great benefit would accrue to the profession. Three years have now elapsed, and my expectations have not been realized: yet nothing tends more to advance obstetrical medicine than the relation of what has occurred in extensive practice at a public charity! Where the practitioner has it in his power to collect a multitude of facts, each of which possesses some share of interest, he owes it to the governors, through whose liberality he is enabled to minister professional relief to his patients, to communicate to his brethren the result of his exertion.

With this view, I take the liberty of submitting a second report of the obstetrical branch of the Westminster General Dispensary, which will embrace the practice of 1819, and may serve as an appendix to my former report.

It is my intention to give, in the like manner, the report of every other successive year; and I am glad to avail myself of the widely-circulated medium of this Journal for so doing; since I can, through it, communicate whatever additional information I possess to those who have seen the volume I published in 1819.

In the following report, as in the former, the reader will find an account of the number of pregnant women delivered in the course of the year 1819, in consequence of recommendations from the governors of the Dispensary. A statement is, also, given of the total number of children born during the same period, as they appear on the registers of the Institution. Cases presenting any particular feature of interest, will be detailed at some future period. Some have occurred that are perfectly new; others, again, which are only interesting in as far as they serve to confirm or illustrate well-known canons in practice. Either class of cases will be found useful; for an accoucheur can never read too often, nor reflect too long over many such cases. They serve to familiarize him with all the probable varieties of labour, and to bring, speedily, to his memory the means of affording assistance in those "puzzling occasions," as an ingenious contemporary once observed, "in which a book like Dr. Conquest's would be of infinite service in the lying-in room."

That part of the report which relates to the use of instruments or to manual labours, has been drawn up with the utmost precision, and is copied verbatim from the register, as well as from the returns of the midwives.

I have again directed a considerable portion of my attention to the subject of miscarriage; nor do I find that the many curious remarks and calculations inserted in my first report under this head, are invalidated by subsequent, and more extensive, information.

In regard to the classification of labours, and that of abortions, which I proposed three years ago, I am happy in the opportunity of observing, that I find them perfectly to suit the exigencies of practice, and to stand the test of experience. Their simplicity, in opposition to the more complicated arrangement in present use, has been found fault with; as if nothing in medical science could be adopted that is not pedantic and periphrastic. I feel confident that, when personal jealousies shall have subsided, what is really useful will be adopted, in spite of selfish considerations.

The assistance which the Westminster General Dispensary affords to poor married women, during their confinement, is not the only benefit those poor females derive from that Institution. Many and complicated are the disorders to which they are exclusively subject, and which require the practical knowledge and assiduous care of the physician-accoucheur. Of this class of diseases, numerous cases have been admitted during the year 1819, many of which have proved highly interesting, as well on account of their nature, as from their successful termination.

A. *Number of Admissions at the Westminster General Dispensary, in the Department of Midwifery.*

During the year 1819, the number of patients under my care were as follow:

| | |
|---|---|
| Pregnancies | 687 |
| Female and children's complaints | 121 |
| Remaining from the preceding year | 77 |
| Total | 885 |

B. *Result of the Cases of Pregnancy.*

| | At the full Time. | | Before the 9th Month. | | Total. |
|---|---|---|---|---|---|
| | Alive. | Still-born. | Alive. | Still-born. | |
| Boys | 382 | 12 | 1 | — | 395 |
| Girls | 283 | 7 | 5 | 2 | 297 |
| General Total | 665 | 19 | 6 | 2 | 692* |

Proportion of boys to girls ---------------- 3 to 2.26

Proportion of children still-born { Boys ------ 1 in $32\frac{11}{12}$; Girls ------ 1 in 33 }

General proportion of still-born children ------ 1 in $32\frac{20}{21}$.

Five women out of the 687 had twins, and produced

Children alive, and at the full time ------------ 9

Still-born, or before the nine months ---------- 1

Proportion of twin cases ---------------- 1 in $137\frac{2}{5}$.

Sex of the twins: { both males in three cases. both female in one case. male and female in one case. }

* Cases of miscarriages under the sixth month, admitted at the Dispensary, are not included.

C. *Nature of the Labours, classed according to Dr. Granville's new Arrangement.*

| | A. | B. | |
|---|---|---|---|
| ACTIVE LABOURS* | Within the first 12 hours, 529. | Beyond 12 hours, 133. | Total, 662. |
| PASSIVE LABOURS† | Manual, 20. | Instrumental, 5. | Total, 25. |
| | | Grand total---------- | 687. |

D. *Enumeration of the Manual and Instrumental Labours.*

The number of passive labours during this year has been small; but it bears a just proportion to that reported on a former occasion. I shall give the details of them in the same tabular form I then adopted, in order to shew the relation which exists between the occurrence of such labours and the particular presentation of the fetus. In none of these labours are the midwives suffered to interfere; so that whatever information is to be found in this part of the report, may be relied on for its accuracy, as the minutest account has been kept of each case that I attended personally.

| *Specification of the Nature of the Labour.* | *Terminated with the Hand.* | *Terminated with Instruments.* Forceps. | *Terminated with Instruments.* Perforator. | *Proport. of the Presentation to the 687 Labours.* |
|---|---|---|---|---|
| The vertex of the head presenting | .... | 1 | ...... | 1 in 687 |
| Head and hand ............... | 1 | .... | ...... | 1 in 687 |
| Arm ...................... | 1 | .... | ...... | 1 in 687 |
| Face ..................... | 1 | 1 | ...... | 1 in $343\frac{1}{2}$ |
| Nates ..................... | 2 | .... | ...... | 1 in $343\frac{1}{2}$ |
| Feet ...................... | 6 | 1 | ...... | 1 in $98\frac{1}{7}$ |
| Umbilical chord ............ | 1 | .... | ...... | 1 in 687 |
| Head impacted ............. | .... | 1 | ...... | 1 in 687 |
| Convulsions (head) ........... | .... | 1 | ...... | 1 in 687 |
| The placenta over the os uteri .. | 1 | .... | ...... | 1 in 687 |
| Hemorrhage,—either from the situation of the placenta; the partial detachment of the same, before or after labour; or after the completion of it | 7 | .... | ...... | 1 in $98\frac{1}{7}$ |
| Total ........ | 20 | 5 | ...... | ...... |
| Cases of retained placenta ...... | 4 | Proportion of Forceps cases, 1 in $137\frac{1}{3}$. | Proportion of Perforator cases, 0. | Proportion of Retained Placenta, 1 in $171\frac{3}{4}$. |
| Total number of labours which required my assistance during the twelve months ......... | 29 | | | |

* Terminated, without the slightest interference, by nature alone.

† When nature becomes *passive*, or insufficient, and the assistance, either of the hand or instruments, is absolutely necessary to terminate the labour.

Proportion of manual labours ---------- 3 in 103$\frac{1}{20}$,
——————— instrumental labours ------ 1 in 137$\frac{1}{3}$,
General proportion of passive labours---- 4 in 110,
compared to the number of aggregate labours.

E. *Mortality.*

In calculating the mortality amongst the lying-in women of a Dispensary, there are several considerations which naturally offer themselves to our attention. In the first place, we have it not in our power, as is the case in private practice, to interfere opportunely with the labour, in order to render that safe which, when neglected, becomes dangerous: on the contrary, our assistance, as I have already observed, is often required too late, and when exhausted nature seems to place the case beyond the reach of art. In the next place, a great many of the patients are in the utmost state of wretchedness and want, so as not to be able to meet the exigencies of their recovery from lying-in; besides being often confined in small, cold, and damp rooms, either in cellars or garrets. Lastly, the dispensary patient is not so immediately under the control of the physician as the hospital patient; nor so much under his vigilant interference as in private practice; so that, very often, his advice is neglected —his injunctions over-ruled—and the timely use of medicines rejected.

| | | |
|---|---|---|
| DIED | After active labours......... | 2 |
| | After passive labours ...... | 2 |
| | Total | 4 |

General proportion of deaths in the aggregate number of labours ------------------------------------ 1 in 171$\frac{3}{4}$.

Both cases of death, after active labour, were from decided phthisis. In one of the cases of death, after passive labour, the patient had been delivered with the forceps, by a general practitioner, before my arrival, in consequence of convulsions. In the other case, I turned the child; the woman having suffered great loss of blood for some days previously, owing to the implantation of the after-birth over the os uteri.

F. *Age of the Patients.*

The age of the women in a state of pregnancy, who have applied at the Dispensary in the course of the year, forms a curious and interesting object of investigation. If we possessed many records of this kind, we might, perhaps, be enabled to ascertain, with more precision than has hitherto been done, the epoch at which women cease to be susceptible of child-bearing; and the age at which they are most prolific. The records of the

Westminster General Dispensary of the last as well as of the present year, when they shall have been multiplied by successive observations, will furnish, in this respect, some facts worth preserving.

Of the 641 pregnant women, whose ages could be ascertained, there were—

| | *Age.* | *Number.* | *Proportion.* |
|---|---|---|---|
| Inclusive | From 16 to 20 | 22 | 1 in $29\frac{3}{22}$ |
| | —— 20 to 30 | 342 | 1 in $1\frac{38}{37}$ nearly |
| | —— 30 to 40 | 238 | 1 in $2\frac{1}{3}$ |
| | —— 40 to 50 | 39 | 1 in $16\frac{1}{2}$ nearly |
| | Average age $30\frac{9}{641}$ | Total 641 | |

The collective age of the above 641 women being 19,239 years.

G. *Abortion.*

It has long been the practice amongst writers on midwifery to assert, that abortions are more frequent in the highly civilized, perhaps luxurious, scenes of life, than in those situations which might be presumed to be most unfavourable to the sex. They have openly stated, and that repeatedly, that, amongst the lower ranks of life, abortions, except from violent external accidents, rarely happen: nay, they have gone so far as to believe, upon what reasons it is difficult to say, that women, in a state of nature, would not be more liable to abortion than other female animals. Thus, then, have the progress of civilization, and the honest enjoyments of the comforts of life, been considered as some of the destructive causes of society! Paradoxical as this idea may appear, I felt anxious that its truth or falsehood should rest upon actual observation and experiment; and, as my public situation at the Dispensary furnishes me with the daily means of watching, and examining, these supposed privileged people, "the lower ranks of life," I thought it my duty to collect from them as much authentic information as I could, in reference to this particular object, with a view of laying it before the public, that a judgment might be formed how far the writers in question are correct, or justified, in their assertions.

It will be seen, then, from what follows, and from what has been stated in the former report that, with regard to abortion, no privileged class exists among women; and that the majority of those causes, to which the frequent occurrence of that event must necessarily be ascribed, act equally on the wretched inhabitants of the Seven-Dials, and on the more fortunate inmates of a palace.

In my last report I stated, that, having closely inquired into the number of miscarriages which occur among the married women in the inferior classes of life in London, I had been able to form a precise idea of the frightful extent to which that lamentable occurrence took place. My inquiries during the subsequent year, embraced by the present report, I regret to say, confirm the results obtained from the previous investigation. In the course of the year 1819, I closely examined upwards of five hundred married women, in that class of life which generally attend at a public Dispensary, (out of the 687 who had come under my notice), as to the number of miscarriages they might have had—their nature, period, and cause—from the time of their marrying up to the period of their applying at the Dispensary; and, from their answers, I have obtained the following results, comprised within an average period of ten years:

| | |
|---|---|
| Number of women who have been questioned as to any miscarriages they have had, during a period of ten years | 515 |
| Had miscarried, out of that number, at some period or other of their marriage, within ten years | 147 |
| Proportion of women who had miscarried, and had also borne live children, to those who had borne live children and never miscarried | 1 in $3\frac{1}{2}$ |

The number of miscarriages which occurred, during a period of ten years, among the 147 women above mentioned, were as follows:

| | |
|---|---|
| Women who miscarried | 147 |
| Number of miscarriages | 372 |
| Proportion for each woman | $2\frac{26}{49}$ |

[To be continued in our next.]

